# Sensitivity of Different Cattle Breeds to the Infestation of Cattle Ticks* Amblyomma variegatum, Rhipicephalus microplus*, and* Hyalomma *spp. on the Natural Pastures of Opkara Farm, Benin

**DOI:** 10.1155/2018/2570940

**Published:** 2018-03-25

**Authors:** Roland Eric Yessinou, Camus Adoligbe, Yao Akpo, Justin Adinci, Issaka Youssao Abdou Karim, Souaïbou Farougou

**Affiliations:** ^1^University of Abomey-Calavi (UAC), Polytechnic School of Abomey-Calavi (EPAC) Production and Animal Health Department, Applied Biology Research Laboratory (LARBA), 01 P.O. Box 2009 Cotonou, Benin; ^2^Laboratory of Ecology, Health and Animal Production, Faculty of Agronomy, University of Parakou, P.O. Box 123 Parakou, Benin

## Abstract

A study was carried out on the Opkara (Benin) cattle farm on 64 cattle of four different breeds (16 individuals per breed) from June to December 2016. During this study, three tick species were found in different numbers,* Amblyomma variegatum* (732),* Rhipicephalus microplus* (8079), and* Hyalomma *spp. (208), with parasitic intensity of 11.90, 126.23, and 3.25, respectively. The interracial comparison of the tick infestation between the cattle showed a significant difference (*P* < 0.001). However, Girolando was more infested than all the cattle breeds. Infestation of* A. variegatum*,* R. microplus*, and* Hyalomma *spp. on the Girolando was, respectively, 19.43 ± 2.71, 171.25 ± 23.50, and 7.12 ± 0.63, but the Borgou were less infested. Borgou breed females were more infested by* A. variegatum* (4.41 ± 1.14) than females Girolando (4.20 ± 0.90). The Crossbred and Azawak females were less infested (*P* < 0.01). The mean of* A. variegatum* on Borgou, Azawak, Crossbred, and Girolando calves was 1.29 ± 0.35, 0.66 ± 0.26, 1.37 ± 0.37, and 2.25 ± 0.48 (*P* < 0.01), respectively. The results of this study can be exploited to include genetic and nongenetic approaches to tick control.

## 1. Introduction

Benin is an agricultural country and livestock play a predominant role in agricultural production [1]. In addition to meat and milk production, cattle are used by farmers to plow the land. The performance of these cattle is negatively affected by ectoparasites. For instance, tick-borne diseases have significant impact on animal productivity; they cause mortality and enormous economic losses for livestock farmers [2]. The acaricides used by breeders for ticks management are not always effective [3]. Chemicals used have a serious impact on the environment and on the safety of meat and milk products that are consumed [4]. Also, access to chemical treatments is not affordable for the poorest breeders. Ticks, especially* Rhipicephalus microplus*, have developed resistance against most of the acaricides used for their control [3]. Many in vitro studies on the effect of the mixture of essential oils and plant on engorged female of* R. microplus* have shown satisfactory results. However, on field essay needs to be conducted to extend such results [5]. Thus, the only rapid control method available now to breeders is the application of synthetic acaricidal molecules [6]. To control these parasites, other control methods can be explored such as the selection of tick-resistant cattle. Studies done on Nkedi Zebu and Ankole exposed to tick infestation have shown intraracial differences in tick load [7], and a difference in infestation was noted according to the cattle ages. The works done by Mattioli et al. [8] on N'Dama, Gobra Zebu, and their crossbred products showed an interracial difference of tick infestations, suggesting that the selection of the cattle can be also based on ticks resistance. Animals selected for their greater resistance to* R. microplus* consistently exhibited a lesser load than their congenerics in whom a resistance criterion was not taken into account [9]. The results obtained by Piper et al. [10] on ticks confirm that the expression of certain genes in the skin of bovines reduces the infestation of the tick of the cattle* R. microplus*. Other studies show that cattle* Bos indicus* have developed resistance against* Amblyomma variegatum* [11]. In any case, the selection of tick-resistant animals can reduce the devastating effect of tick infestation. This method of control is not harmful to the environment, entails no additional costs, and can be a viable solution for the livestock sector. On the other hand, in most cases, ticks and vectors of trypanosomiasis are found on the same pastures. The selection of resistant animals is not only for tick management but also for limiting the tick resistance to acaricides and break down the intensive use of synthetic products for these ectoparasites' control. This study aims at assessing the level of infestation of different tick species in the same environment as well as the influence of breeds and sex.

## 2. Materials and Methods

### 2.1. Study Area

**Figure 1:**
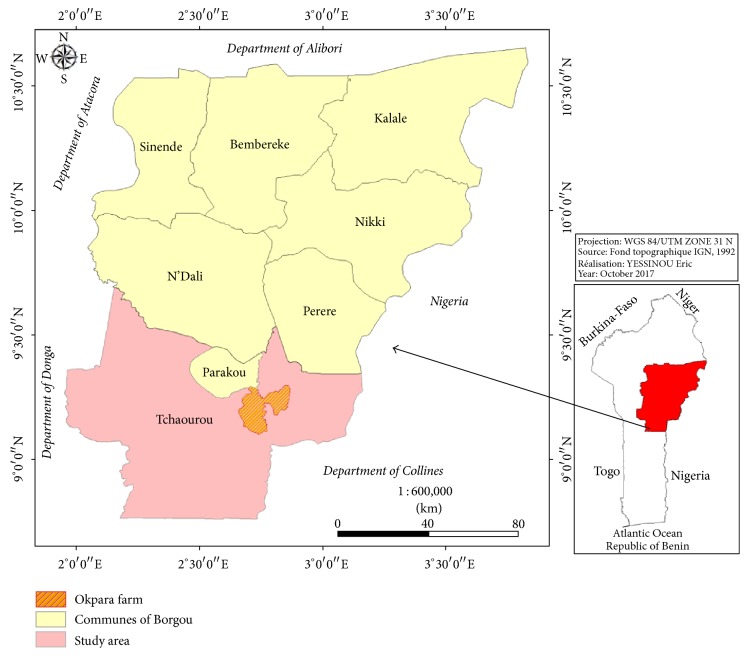
The map of Benin showing the district (Okpara) where experiment on cattle breed was carried out.

The works were carried out in the communes of Tchaourou northeast of Benin. This locality belongs to the agroecological zone (Cotton Zone of Center Benin or Zone V). The commune of Tchaourou is located in the department of Borgou at 11°08′ north latitude and 2°56′ east longitude. It has an area of 7,256 km^2^; it represents 28% of the total area of this department and about 6.5% of the national territory. It is bounded on the north by the municipalities of Parakou, Pèrèrè, and N'Dali, on the south by the commune of Ouèssè, on the east by the Federal Republic of Nigeria, and on the west by the municipalities of Bassila and Djougou. This strategic geographical position is undoubtedly a major asset to be exploited by the municipal authorities for the promotion of the local economy. Like the other communes of the department of Borgou, the commune of Tchaourou is subject to the influence of the South Sudanese climate. It is a modal united climate characterized by a dry season and a wet season. Rainfall totals vary between 1100 and 1200 mm/year and range from 6 to 7 months wet during the year. The ecological preferences of ticks are variable, as each species needs specific environmental conditions enabling it to live in a given biotope, which influences its geographical distribution. Several ecological factors influence the survival and development of ticks, particularly temperature, relative humidity, and vegetation cover. This pluviometry distribution may, therefore, favor the emergence of certain species of the tick in this commune (Figure 1).

### 2.2. Tick Collection

The study carried out took into account the four different cattle races existing on the Opkara breeding farm. A total of 64 animals (4 bulls, 4 cows, 4 calves, and 4 velles by breed) were randomly selected from the animals to serve as a study sample. The animals were reared on the same holding and were brought in the same natural pasture. Tick sampling was carried out weekly on each animal for 20 weeks from July to December 2016, a period of rainy season and dry season. The animals were kept in the contention corridors to collect ticks in the anatomical region. Acaricides were not used during the two weeks prior to the experiment and during its duration. The dermatological lesions as well as the other, clinical signs observed on the animals were noted but we did not make the case in this study. The animals consist of Borgou (Somba or Lagunaire × Zebu White Fulani), Azawak (Zebu), Crossbred (Borgou × Girolando), and Girolando (Gyr × Holstein).

### 2.3. Conservation and Identification of Ticks

Ticks were collected manually from each animal using pincers on the seven predefined parts (head and neck, ears, back and croup, abdominal, ventrogenital, tail, and legs) on each animal by a team after its restraint. The ticks were stored in seven vials containing 70% ethanol and labeled according to the predefined part of each animal. Another label with necessary information was inserted directly into each vial having sample before closing completely. The information was marked in pencil and includes the date of collection, animal identification number, and other relevant data. The species identification was carried out using the identification key elaborated by Walker et al. [12] with an electronic binocular microscope (Olympus) at 100x magnification.

### 2.4. Statistical Analysis

The data was saved in Excel 2007; the R software was used to analyze the data. The abundance and mean parasitic intensity were calculated. For the mean number of ticks per animal, the factors of variation considered were tick species, bovine breed, tick life stage, cattle anatomy region, and cattle infestation index. For each variation factor, the *F* test was used to determine its significance and the means were compared two by two using Student's *t*-test. Evaluation of ticks load was as follows:

- Abundance (*A*) is the ratio of the total number of individuals of a parasitic species (*n*) to the total number of individuals examined (*H*). *A* = *n*/*H*.
- The mean parasitic intensity (*I*) corresponds to the ratio of the total number of individuals of a parasitic species (*n*) in a host sample to the number of infested (*N*) hosts in the sample. *I* = *n*/*N*. For the mean parasitic intensities (*I*), the classification adopted is that of Bilong-Bilong and Njiné [13]:
  - *I* < 10: mean parasitic intensity is very low,
  - 10 < *I* < 50: mean parasitic intensity is low,
  - 50 < *I* < 100: mean parasitic intensity is average,
  - *I* > 100: mean parasitic intensity is high.

## 3. Result

### 3.1. Abundance and Mean Parasitic Intensity for Tick Species

A total of 9049 ticks were collected, and the tick species encountered in the study area were* A. variegatum*,* R. microplus*, and* Hyalomma *spp. The number of ticks by species, their abundance, and the mean parasitic intensity are described in Table 1.

### 3.2. Infestation Index of the Tick's Species Parasite of Cattle

The Girolando infestation index by* R. microplus* is three times higher than that of the Borgou. This latter represents the lowest index in contrast to the infestation index by* A. variegatum* which was 2.20 in Girolando followed by Borgou which was 1.39 (Table 2).

### 3.3. Influence of Breed Cattle on Tick's Different Species Infestation

The effect of the cattle breed on tick infestation showed that Girolando cattle are more infested (*P* < 0.01) by ticks* A. variegatum* (19, 43) compared to breeds Borgou (12.31), Azawak (8.25), and Crossbred (7.68), which all have similar rates of infestation. Likewise, infestations of* R. microplus* in cattle of the breeds Borgou (62.43), Azawak (98.81), and Crossbred (102.56) are lower than Girolando (171.25) (*P* < 0.001). In addition, infestation of tick* Hyalomma *spp. was high on Girolando breed (7.12) compared to that of the Borgou (2.31), Azawak (1.18), and Crossbred (1.93) (Table 3).

### 3.4. Influence of Cattle Breed on Infestation of Different Stasis of Tick

Imaginary stasis is as follows: infestation means of Borgou, Azawak, Crossbred, and Girolando by* A. variegatum* and* R. microplus* females are significantly different (*P* < 0.001) with the highest average on Girolando, but no significant difference was noted on male ticks. However, with* Hyalomma *spp., a significant difference was reported with infestation mean. Preimaginary stasis is as follows: mean infestations of Borgou, Azawak, Crossbred, and Girolando cattle by* A. variegatum*,* R. microplus*, and* Hyalomma *spp. are significantly different (*P* < 0.001). The nymphs of ticks found on the body of Girolando are more numerous than those of the other races in our study (Table 4). In general, it appears from this analysis that the* R. microplus* species was the most abundant and the most infesting to all stasis and that on the Girolando cattle. The males of* A. variegatum* and* R. microplus* were more present on Azawak cattle than other breeds. However,* Hyalomma *spp. were less infesting than other tick species.

### 3.5. Infestation of Anatomical Regions of Cattle by Different Tick Species

The mean of the tick species collected in the anatomical regions of the cattle breeds is presented in Table 5. On head and neck, the mean infestation of tick's* R. microplus* is significantly different (*P* < 0.001) with the highest average noted on Girolando. However, the mean of* Hyalomma* spp. on the cattle breeds has been significantly different at the 5% (*P* < 0.05). Cattle ears have been also infested by* A*.* variegatum, R. microplus*, and* Hyalomma *spp. but* R. microplus* is the highest mean of infestation and was found on the Girolando (*P* < 0.001). The mean of infestation* R. microplus* on the back and rump of Borgou is 4.21 ± 0.78. It represents the lowest infestation of this part of the body, but the highest infestation is noted on the Girolando (7.84 ± 1.06) (*P* < 0.05). On the Girolando the level infestation of* A. variegatum* on the back and the rump has been of 1.25 ± 0.26 followed by Borgou (0.93 ± 0.24) (*P* < 0.01). At level of the abdominal region, no significant differences exist between the mean of* A. variegatum* populations collected from the different cattle (*P* > 0.05). Nevertheless, in the two other tick species,* R. microplus* and* Hyalomma *spp., a significant difference was noted on the infestation of cattle. Girolando (9.68 ± 1.16) and Azawak (6.62 ± 1.03) were more infested by* R. microplus* than the other two races. The Girolando are more infested by the* Hyalomma *spp. (1.06 ± 0.27) than the Borgou (0.37 ± 0.13). Ticks* A. variegatum* that were collected in the ventrogenital region were more numerous in Girolando (2.09 ± 0.39) than in Borgou (1.28 ± 0.36) (*P* < 0.05). The preference of* R. microplus* for the ventral genital region appears to be similar of the Azawak (18.21 ± 3.73) and the Crossbred (18.28 ± 3.15), but the Girolando (33.87 ± 6.70) are more infested. The presence of ticks was also noted on the cattle tail prospected. The mean of* R. microplus* was 12.28 ± 2.09, 10.43 ± 2.01, 8.50 ± 1.76, and 4.96 ± 0.91, respectively, in the Girolando, Crossbred, Azawak, and Borgou (*P* < 0.05). A lower infestation of* Hyalomma *spp. was noted on the tail of different races; however, they were high with Girolando (0.31 ± 0.09) followed by Crossbred (0.12 ± 0.08). These ticks were present at the same rate in the other two races. The* A. variegatum* ticks were found on cattle in varying degrees (*P* < 0.05). Mean of* R. microplus* observed on the legs of Borgou, Azawak, and Crossbred is similar but for Girolando (*P* < 0.05) was highly infested.* A. variegatum *and* Hyalomma *spp. were also found on the legs of cattle. The mean number of ticks per anatomical region calculated from the mean of ticks harvested in the study area showed that Girolando cattle are more attracted to ticks than other cattle breeds. It should also be noted that the anatomical regions (head and neck, ears, ventrogenital, and tail) are more infested with* R. microplus* in Girolando and other cattle. The Borgou are less infested by ticks than the Girolando, Crossbred, and Azawak (Table 5).

### 3.6. Influence of Sex of Cattle on Tick's Infestation

Infestation of each tick species according to the age and sex categories of the animals was also the subject of this study. Borgou breed females were more infested by* A. variegatum* (4.41 ± 1.14) than Girolando females (4.20 ± 0.90). The Crossbred and Azawak females have been less infested, respectively, in the order of 1.79 ± 0.42 and 1.33 ± 0.38 (*P* < 0.01). In addition, no significant difference was noted between the infestation means of Borgou, Azawak, Crossbred, and Girolando males. The mean of* A. variegatum* on Borgou, Azawak, Crossbred, and Girolando calves was 1.29 ± 0.35, 0.66 ± 0.26, 1.37 ± 0.37, and 2.25 ± 0.48 (*P* < 0.01), respectively. Girolando breeds have been more infested (1.75 ± 0.36), followed by Azawak (1.00 ± 0.25). Borgou breeds were the least infested by* A. variegatum* (*P* < 0.001). Tick* R. microplus* infestation was greater in male Girolando, Azawak, and Crossbred bovines with the respective mean of 54.79 ± 19.66, 38.62 ± 12.99, and 32.54 ± 10.57 than male bulls Borgou 25.37 ± 6.84. Ticks* R. microplus* collected from the Borgou, Azawak, Crossbred, and Girolando females have been in the order of 17.79 ± 5.22, 21.37 ± 6.74, 21.87 ± 7.86, and 39.79 ± 13.38 (*P* > 0.05), respectively. In the calves also no significant differences were noted in infestation with* R. microplus* (*P* > 0.05). The infestation of the Girolando breeds was 21.87 ± 6.65 compared to 1.29 ± 0.42 on the Borgou breeds and has been the least abundant infestation followed by Azawak 5.12 ± 1.85 (*P* < 0.01) (Table 5).* Hyalomma* spp. ticks were less important on Azawak (0.12 ± 0.06) and Crossbred (0.25 ± 0.09) than Girolando (1.16 ± 0.30) and Borgou (0.54 ± 0.15) males (*P* < 0.001). The cows of the four breeds were also exposed to the infestation of* Hyalomma *spp. (*P* < 0.01) and calves (*P* < 0.001) and velles (*P* < 0.05) (Table 6). From this analysis, we note a great infestation of tick's* R. microplus* on the farm during our study. Males of Girolando, Azawak, and Crossbred race are the most exposed as well as females. The calves and calves of the Borgou were less infested by* R. microplus*.

**Figure 2:**
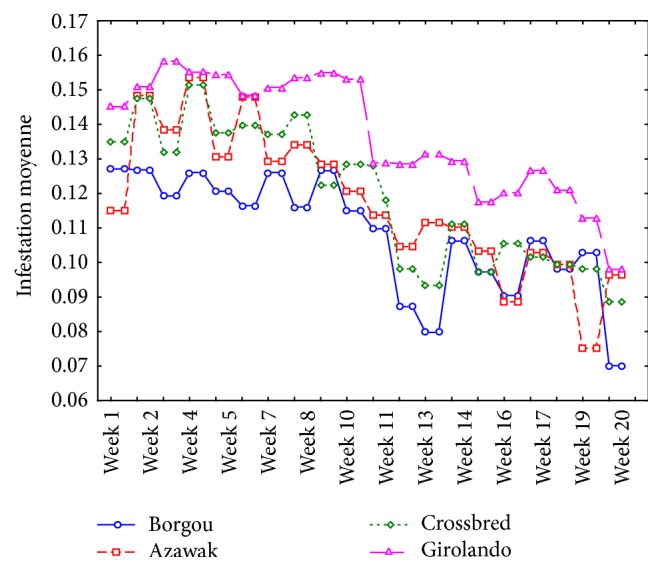
Relative attraction of cattle on ticks (*Amblyomma variegatum*,* Rhipicephalus microplus*, and* Hyalomma *spp.). Mean infestation = [log⁡(tick  counts + 1)]/number of animals of the breed.

Tick parasitic charge was influenced by time and season. A highest attractiveness was observed during the first ten (10) weeks which correspond to the rainy season, period of tick abundance. But, during the last ten (10) weeks, a drop in attractiveness was noted. The highest attractivity was noted in the Girolando and lower in the Borgou (Figure 2).

## 4. Discussion

In this study, 9049 ticks were collected and three tick's species were identified, including* R. microplus*, which was the most abundant (89.28%) with 126.23 as mean parasitic intensity. This revealed a heterogeneous distribution, reason of its parasitic ubiquity. The mean parasitic intensity is high (*I* > 100), because the tick can be found on cattle at all stages of development, including larvae, nymph, and adults. In addition, it was a monotonic monophasic species with strong parasitic selectivity for Girolando. It has also a very short breeding cycle and a high ecological adaptation capacity [14]. On the other hand,* A. variegatum*, despite its capacity to infest the cattle, has a lower mean parasitic intensity (10 < *I* < 50). The same observation was made with* Hyalomma* spp. whose mean parasitic intensity was very low *I* < 10. These ticks have a development cycle that takes place on several hosts and they are less invasive than* R. microplus* [15]. Although consistent with other findings [16], our results showed opposite tendency compared to what was reported few years ago with regard to tick species distribution and abundance around Benin. In fact,* A. variegatum* was the most abundant and widely distributed species in northern Benin [17]. Several authors have, however, revealed an abundance of* A. variegatum *in cattle farms [18]. Studies carried out in northern Benin prior to the arrival of the Girolando on the Opkara breeding farm did not mention the presence of* R. microplus* [17].* R. microplus* was accidentally introduced in Benin particularly on Kpinnou farm and later on Opkara farm through the importation of Brazilian Girolando cattle [19]. Opkara farm constitutes a pole of diffusion of* R. microplus* in the North of Benin. Actually* R. microplu*s is hydrophilic and develops easily in humid climate. Hence the environmental conditions at Opkara (rainfall higher than 1200 mm and mean annual temperature varying between 26 and 27°C) are favorable to the evolution of this tick. Due to the lack of grassland in the study area, cattle are taken to the surrounding woody forest for grazing which increases contact between ticks and animals as the existing microenvironment in woody forest is highly favorable for tick development [20]. This may justify the high infestation rate noted. In this study, index of infestation was used to assess the attraction exerted by each animal on the ticks. The observed variability between animals in the four breeds suggests that ability to attract ticks may be directly linked to genetic factors and indirectly linked to environmental factors and herd management. Animals at the head or tail of the herd are less exposed to ticks than those in the middle of the herd. This may influence infestation rate in between breeds as the position of individual breeds when grazing is not standard. The introduction of* R. microplus* completely changed the pattern of ticks infestation in Benin [18]. The infestation of different animals by* R. microplus* showed a large intraracial variation (*P* < 0.001). The Borgou cattle were the most resistant and the Girolando cattle the least resistant to ticks. It was clear that tick resistance varies not only from one anatomical region to another, but, more markedly, from one breed to another. The Borgou have the smallest mean tick infestation for all ticks species including* A. variegatum*,* R. microplus*, and* Hyalomma *spp., therefore less susceptible than all other breeds of cattle. Borgou cattle are crossbreeding product of the short-horned bulls of West Africa (Somba or Lagunaire) and the Zebu White Fulani* (Bos taurus)* x zebus* (Bos indicus)* [21]. Previous studies have shown that taurine* (Bos taurus)* and zebu* (Bos indicus)* were tick-resistant [22]. Hence this may explain why Borgou cattle are less sensitive to ticks infestation. Bulls are again in Australia, and zebu were imported because of their resistance to ticks and their adaptation to heat. They were used to increase the performance of previously introduced bulls [23]. Bulls are tick-resistant as they release 90% of the larvae after scratching after 24 hours [24]. During taurine infestation by tick larvae, histamine released by mast cells is transported to the site of attachment of the tick by eosinophils. It is noted that the resistance of the taurine is correlated to the concentration of eosinophils at the site of attachment of the tick. Eosinophils therefore play a role in tick rejection by concentrating histamine released by mast cells at the tick attachment point [25]. The concentration of this mediator at the cutaneous level is correlated to the scratching behavior on the part of the host [26]. In Australia, zebu have been imported because of their resistance to ticks and their adaptation to heat. They were used to increase the performance of previously introduced bulls [23]. Borgou was being a cross between taurine and zebu, their response to tick infestation was linked to the presence of a high percentage of the zebu/taurine gene in their organisms. African cattle are more resistant to ticks than exotic bovines [24]. This is consistent with our results as in this study Girolando cattle, which were also crossbred products of Gyr* (Bos indicus)* and Holstein* (Bos taurus)* were more susceptible to ticks than other local breeds of cattle. The works carried out by Jonsson et al. [27] showed a low level of resistance of Holstein-Friesian breed to ticks, particularly* R. microplus*, which usually results in high morbidity and mortality [28]. The Girolando cattle imported to Benin are facing adaptation issues (thermal stress, climatic phenomena) and nutritional deficiencies that weaken their organism and make them vulnerable to ticks. The other two breeds taken into account in this study are Borgou x Girolando products and Azawak cattle. Azawak cattle are originally from Niger and are known for their resistance to heat. These two breeds are less infested than Girolando cattle. The number of ticks counted per body region varied significantly. The head, neck, and ventrogenital region of Borgou and Girolando cattle have the greater number of* A. variegatum*. However,* R. microplus* was abundant at the head, ears, and tails as well as the ventrogenital regions. Our results were similar to those obtained by Awa et al. [29] who have shown a preference for ticks in the ventrogenital and abdominal region. The relation between tick rostrum size and infested region was reported by Farougou et al. [17]. In this research, majority of* R. microplus* was found in the ventrogenital region of Borgou, Azawak, Crossbred, and Girolando (*P* < 0.01).* R. microplus* prefers thin-skinned regions because it is brevirostre. However,* A. variegatum* and* Hyalomma *spp. can be seen everywhere. This finding is in line with that of Barre and Uilenberg [30]. The difference of infestation may be associated with competition between different tick species; for example, the tick* Rhipicephalus microplus* is an invasive species that has higher ecological adaptability capacity and can gradually replace other ticks species [18]. This study also revealed the influence of animal age on tick infestation. Calves are less infested than bulls and cows. These results were similar to those obtained by Matzigkeit [31], which show that young cattle were more resistant to ticks than older ones and young [32]. It should also be noted that the management of young cattle can contribute to the reduction of infestation. They were sometimes kept in a stall, reducing the risk of their exposure to ticks in pastures. The parasitic load of male's cattle was higher than that of the females. This difference can be explained by the stimuli and the tick tropism. The amount of carbon dioxide (CO_2_) emitted is the first determining factor in the detection of the presence of the host by the ticks [33]. Since this quantity was proportional to the size of the host, male bovines emit much more gas than the female bovine animals, which explains the high parasitic load found at their level [34]. These results were contrary to the results reported by Gharbi et al. [35] which show that female cattle were more infested than males. However, they agree with the observations of Chartier et al. [15], who reported that ticks were usually more frequent on bulls than on cows. It would be beneficial to take into account all these factors in the selection of breeds to reduce the damage by the ticks to infested animals.

## 5. Conclusion

Ticks, due to their direct impact and the diseases they transmit, are one of the major constraints to the development of cattle breeding in northern Benin. The study carried out in the locality of Opkara on the infestation of cattle by ticks allowed us to identify three endemic species of ticks including* A. variegatum*,* R. microplus*, and* Hyalomma *spp. Girolando cattle are the most sensitive to all ticks species. Abundance of* R. microplus* once again testifies its adaptability and invasiveness. It may spread all over the country and even reach neighboring countries. Taking into account the information obtained from this work, it would be possible to integrate genetic approaches to tick control in order to improve the health management of cattle herds.

## Figures and Tables

**Table 1:** Abundance and mean parasitic intensity for tick's species.

Ticks species	Number of ticks	Abundance (%)	Mean parasitic intensity (*I*)
*A. variegatum*	762	8.42	11.90
*R. microplus*	8079	89.28	126.23
*Hyalomma *spp.	208	2.29	3.25

Mean parasitic intensity (*I*) corresponds to the ratio of the total number of individuals of a parasite species (*n*) in a sample of hosts on the number of infested hosts (*N*) in the sample. *I* = *n*/*N*.

**Table 2:** Infestation index of the tick's species parasite of cattle.

Cattle	Infestation index^1^		
	*A. variegatum*	*R. microplus*	*Hyalomma *spp.
Borgou	1.39	8.14	0.26
Azawak	0.93	12.82	0.13
Crossbred	0.87	13.20	0.22
Girolando	2.20	23.02	0.81

^1^Infestation of the animal/mean infestation of the herd.

**Table 3:** Influence of breed cattle on tick different species infestation.

Tick species	Borgou	Azawak	Crossbred	Girolando	Sig
	Means ± SE	Means ± SE	Means ± SE	Means ± SE	
*A. variegatum*	12.31 ± 3.30^b^	8.25 ± 1.40^b^	7.68 ± 1.10^b^	19.43 ± 2.71^a^	*∗∗*
*R. microplus*	62.43 ± 13.15^b^	98.81 ± 16.76^b^	102.56 ± 9.11^b^	171.25 ± 23.50^a^	*∗∗∗*
*Hyalomma *spp.	2.31 ± 0.47^b^	1.18 ± 0.34^b^	1.93 ± 0.30^b^	7.12 ± 0.63^a^	*∗∗∗*

SE: standard error; ^*∗∗*^*P* < 0.01; ^*∗∗∗*^*P* < 0.001. a, b: means with the same letters within lines are not significantly different, *P* > 0.05 (averages of the same line, followed by the same letter, do not differ significantly at the 5% level).

**Table 4:** Influence of cattle breed on infestation of different stasis of tick.

Ticks species	Stasis	Borgou	Azawak	Crossbred	Girolando	Sig
		Means ± SE	Means ± SE	Means ± SE	Means ± SE	
*A. variegatum*	Males	1.96 ± 0.46^a^	2.59 ± 0.79^a^	1.53 ± 0.32^a^	2.4 ± 0.46^a^	NS
	Females	2.93 ± 0.64^b^	2.00 ± 0.32^b^	2.06 ± 0.39^b^	6.34 ± 1.30^a^	*∗∗∗*
	Nymphs	0.62 ± 0.23^ab^	0.15 ± 0.07^b^	0.25 ± 0.08^b^	0.96 ± 0.25^a^	*∗∗*

*R. microplus*	Males	2.00 ± 0.39^a^	2.40 ± 0.44^a^	2.18 ± 0.40^a^	2.06 ± 0.36^a^	NS
	Females	32.37 ± 5.57^b^	52.93 ± 9.50^b^	55.84 ± 8.22^b^	97.59 ± 13.47^a^	*∗∗∗*
	Nymphs	1.18 ± 0.37^ab^	1.15 ± 0.31^ab^	0.35 ± 0.11^b^	2.06 ± 0.40^a^	*∗∗*

*Hyalomma *spp.	Males	0.46 ± 0.14^b^	0.28 ± 0.09^b^	0.28 ± 0.08^b^	1.15 ± 0.23^a^	*∗∗∗*
	Females	0.59 ± 0.13^b^	0.31 ± 0.09^b^	0.46 ± 0.11^b^	2.15 ± 0.41^a^	*∗∗∗*
	Nymphs	0.12 ± 0.05^ab^	0.00 ± 0.00^b^	0.21 ± 0.07^ab^	0.25 ± 0.08^a^	*∗*

SE: standard error; ^NS^*P* > 0.05; ^*∗*^*P* < 0.05; ^*∗∗*^*P* < 0.01; ^*∗∗∗*^*P* < 0.001. a, b, and ab: means with the same letters within lines are not significantly different, *P* > 0.05 (averages of the same line, followed by the same letter, do not differ significantly at the 5% level).

**Table 5:** Infestation of anatomical regions of cattle by different tick species.

Anatomical regions	Ticks species	Borgou	Azawak	Crossbred	Girolando	Sig
		Means ± SE	Means ± SE	Means ± SE	Means ± SE	
Head and neck	*A. variegatum*	1.37 ± 0.46^a^	0.78 ± 0.16^a^	0.71 ± 0.17^a^	1.84 ± 0.36^a^	NS
	*R. microplus*	5.78 ± 1.08^b^	7.81 ± 1.38^b^	9.78 ± 1.61^b^	16.09 ± 2.29^a^	*∗∗∗*
	*Hyalomma *spp.	0.12 ± 0.06^ab^	0.06 ± 0.04^b^	0.03 ± 0.03^b^	0.28 ± 0.09^a^	*∗*

Ears	*A. variegatum*	1.00 ± 0.30^ab^	0.65 ± 0.14^b^	0.62 ± 0.16^b^	1.68 ± 0.37^a^	*∗*
	*R. microplus*	4.34 ± 0.81^b^	8.46 ± 2.01^b^	6.84 ± 1.29^b^	17.34 ± 3.96^a^	*∗∗∗*
	*Hyalomma *spp.	0.15 ± 0.06^a^	0.12 ± 0.05^a^	0.15 ± 0.07^a^	0.18 ± 0.08^a^	NS

Back and croup	*A. variegatum*	0.93 ± 0.24^a^	0.37 ± 0.08^b^	0.31 ± 0.10^b^	1.25 ± 0.26^a^	*∗∗*
	*R. microplus*	4.21 ± 0.78^b^	4.62 ± 0.98^b^	5.34 ± 0.86^ab^	7.84 ± 1.06^b^	*∗*
	*Hyalomma *spp.	0.28 ± 0.11^b^	0.12 ± 0.05^b^	0.28 ± 0.08^b^	1.06 ± 0.22^a^	*∗∗∗*

Abdominal	*A. variegatum*	0.50 ± 0.24^a^	0.46 ± 0.14^a^	0.50 ± 0.14^a^	1.03 ± 0.25^a^	NS
	*R. microplus*	4.18 ± 0.74^b^	6.62 ± 1.03^b^	5.15 ± 0.80^b^	9.68 ± 1.16^a^	*∗∗∗*
	*Hyalomma *spp.	0.37 ± 0.13^b^	0.12 ± 0.05^b^	0.21 ± 0.09^b^	1.06 ± 0.27^a^	*∗∗∗*

Ventrogenital	*A. variegatum*	1.28 ± 0.36^ab^	1.06 ± 0.25^ab^	0.90 ± 0.22^b^	2.09 ± 0.39^a^	*∗*
	*R. microplus*	9.78 ± 1.99^b^	18.21 ± 3.73^b^	18.28 ± 3.15^b^	33.87 ± 6.70^a^	*∗∗*
	*Hyalomma *spp.	0.12 ± 0.05^a^	0.06 ± 0.04^a^	0.12 ± 0.05^a^	0.18 ± 0.08^a^	NS

Tail	*A. variegatum*	0.68 ± 0.17^a^	0.56 ± 0.12^a^	0.53 ± 0.12^a^	1.21 ± 0.31^a^	NS
	*R. microplus*	4.96 ± 0.91^b^	8.50 ± 1.76^ab^	10.43 ± 2.01^ab^	12.28 ± 2.09^a^	*∗*
	*Hyalomma *spp.	0.03 ± 0.03^b^	0.03 ± 0.03^b^	0.12 ± 0.08^ab^	0.31 ± 0.09^a^	*∗*

Legs	*A. variegatum*	0.25 ± 0.10^b^	0.18 ± 0.08^b^	0.25 ± 0.07^b^	0.62 ± 0.14^a^	*∗*
	*R. microplus*	2.09 ± 0.46^b^	2.65 ± 0.64^b^	2.18 ± 0.51^b^	5.00 ± 0.89^a^	*∗∗*
	*Hyalomma *spp.	0.06 ± 0.04^b^	0.06 ± 0.04^b^	0.03 ± 0.03^b^	0.31 ± 0.11^a^	*∗∗*

SE: standard error; ^NS^*P* > 0.05; ^*∗*^*P* < 0.05; ^*∗∗*^*P* < 0.01; ^*∗∗∗*^*P* < 0.001. a, b, and ab: means with the same letters within lines are not significantly different, *P* > 0.05 (averages of the same line, followed by the same letter, do not differ significantly at the 5% level).

**Table 6:** Influence of sex of cattle on tick's infestation.

Ticks species	Cattle	Borgou	Azawak	Crossbred	Girolando	Sig
		Means ± SE	Means ± SE	Means ± SE	Means ± SE	
*A. variegatum*	Bulls	2.29 ± 0.54^a^	2.50 ± 0.40^a^	1.41 ± 0.60^a^	4.75 ± 1.73^a^	NS
	Cows	4.41 ± 1.14^a^	1.33 ± 0.38^b^	1.79 ± 0.42^ab^	4.20 ± 0.90^a^	*∗∗*
	Calves	1.29 ± 0.35^ab^	0.66 ± 0.26^b^	1.37 ± 0.37^ab^	2.25 ± 0.48^a^	*∗*
	Velles	0.20 ± 0.08^b^	1.00 ± 0.25^b^	0.54 ± 0.24^b^	1.75 ± 0.36^a^	*∗∗∗*

*R. microplus*	Bulls	25.37 ± 6.84^a^	38.62 ± 12.99^a^	32.54 ± 10.57^a^	54.79 ± 19.66^a^	NS
	Cows	17.79 ± 5.22^a^	21.37 ± 6.74^a^	21.87 ± 7.86^a^	39.79 ± 13.38^a^	NS
	Calves	2.95 ± 0.90^a^	10.20 ± 4.19^a^	11.91 ± 5.01^a^	19.16 ± 5.75^a^	NS
	Velles	1.29 ± 0.42^b^	5.12 ± 1.85^b^	11.50 ± 5.04^ab^	21.87 ± 6.65^a^	*∗∗*

*Hyalomma *spp.	Bulls	0.54 ± 0.15^b^	0.12 ± 0.06^b^	0.25 ± 0.09^b^	1.16 ± 0.30^a^	*∗∗∗*
	Cows	0.66 ± 0.14^b^	0.25 ± 0.10^b^	0.37 ± 0.11^b^	1.58 ± 0.50^a^	*∗∗*
	Calves	0.20 ± 0.10^b^	0.16 ± 0.07^b^	0.20 ± 0.08^b^	1.20 ± 0.35^a^	*∗∗∗*
	Velles	0.16 ± 0.13^b^	0.25 ± 0.10^b^	0.45 ± 0.13^ab^	0.79 ± 0.21^a^	*∗*

SE: standard error; ^NS^*P* > 0.05; ^*∗*^*P* < 0.05; ^*∗∗*^*P* < 0.01; ^*∗∗∗*^*P* < 0.001. a, b, and ab: means with the same letters within lines are not significantly different, *P* > 0.05 (averages of the same line, followed by the same letter, do not differ significantly at the 5% level).
